# Comparing data sources in estimating disability-adjusted life years (DALYs) for ischemic heart disease and chronic obstructive pulmonary disease in a cross-sectional setting in Finland

**DOI:** 10.1186/s13690-020-00439-6

**Published:** 2020-06-18

**Authors:** Laura Paalanen, Jaakko Reinikainen, Tommi Härkänen, Tiina Mattila, Tiina Laatikainen, Pekka Jousilahti, Hanna Tolonen

**Affiliations:** 1 Department of Public Health Solutions, Finnish Institute for Health and Welfare (THL), P.O. Box 30, FI-00271 Helsinki, Finland; 2 Department of Pulmonary Diseases, Heart and Lung Center, Helsinki University Hospital, Helsinki, Finland; 3 Faculty of Medicine, University of Helsinki, Helsinki, Finland; 4 Institute of Public Health and Clinical Nutrition, University of Eastern Finland, Kuopio, Finland; 5 Joint Municipal Authority for North Karelia Social and Health Services (Siun sote), Joensuu, Finland

**Keywords:** Disability-adjusted life year, Disease burden, Health examination survey, Self-report, Register data, Ischemic heart disease, Chronic obstructive pulmonary disease

## Abstract

**Background:**

The disability-adjusted life years (DALYs) summarize the burden of years of life lost (YLL) due to premature mortality and years lost due to disability (YLD). Our aim was to estimate the burden of ischemic heart disease (IHD) and chronic obstructive pulmonary disease (COPD) in Finland in 2012, and to examine, how much the YLD are affected by the use of different data sources.

**Methods:**

The YLL were calculated using mortality data for the Finnish 25–74-year-old population in 2012. The YLD were calculated using data from the FINRISK 2012 survey (3041 males, 3383 females aged 25–74 years) and then directly adjusted to the corresponding population. Different administrative registers on 1) hospital inpatient episodes and specialist outpatient visits, 2) entitlement to specially reimbursed medicines, and 3) purchases of prescribed medicines were used for estimation of the YLD in addition to self-reported data. The DALYs were calculated without age-weighting.

**Results:**

The YLL for IHD were 37.5 for males and 9.1 for females per 1000 population among 25–74-year-old people in Finland in 2012. The YLD for IHD varied markedly depending on which data sources were used. All data sources combined, the YLD per 1000 were 5.3 for males and 2.5 for females resulting in estimated 42.8 and 11.6 DALYs per 1000 due to IHD among males and females, respectively. For COPD, the YLL were 4.7 for males and 2.0 for females per 1000. Also for COPD, the YLD varied markedly depending on data sources used. The YLD per 1000 based on all data sources combined were 2.0 for males and 1.6 for females. As a result, estimated 6.7 and 3.6 DALYs per 1000 were due to COPD among males and females, respectively.

**Conclusions:**

Especially for COPD, all mild disease cases could probably not be identified from the included registers. Thereby, including survey data improved the coverage of the data. The YLD of IHD and COPD varied markedly between the data sources used in the calculations. However, as YLL constituted a major part of DALYs for these diseases, the variation in YLD did not lead to substantial variation in DALYs.

## Background

Disability-adjusted life year (DALY) is a summary measure, which combines the burden of both premature mortality and morbidity. One unit of DALY corresponds to one lost year of ‘healthy’ life [1]. For DALYs, years of life lost (YLL) due to premature mortality and years lost due to disability (YLD) are counted and summed up. In the framework of the Global Burden of Disease (GBD) Study, the methods for calculating DALYs have been continuously developed [1–3].

The calculation of DALYs includes several possible sources of error. In the case of the YLL, the calculations are reasonably straight-forward, if reliable data on causes of death are available, and uncertainty over YLL mainly exists due to ill-defined deaths. For YLD, accurate data on morbidity on population level may be less readily available. In Finland, data from many high-quality administrative population registers are available for researchers and widely utilized in public health research [4–7]. However, most health care registers with a long history of systematic data collection, concentrate on hospitalizations, and thereby may not cover persons with a less severe condition or chronic disease diagnosed a long time ago and treated in outpatient clinics or on private sector. Data on the prevalence of health problems which do not need hospitalizations may be identified from other registers, such as entitlement to specially reimbursed medicines or medicine purchases but using these data has also several limitations.

Complementing register data with population-based health examination survey (HES) data may help to fill in possible gaps in registers with more representative prevalence rates as a result. Burden of disease studies are currently dependent on severity data estimating the distribution of severity levels of each condition from asymptomatic to severe state [3]. Therefore, it is crucial that also the less severe cases would be identified to achieve accurate YLD estimates.

Ischemic heart disease (IHD) was the leading cause of YLL in 2017 globally and in the European Union, whereas chronic obstructive pulmonary disease (COPD) ranked as seventh globally and as fifth in the European Union [8, 9]. In Finland, IHD mortality has decreased markedly but was still the leading cause of deaths in 2017, and, more practically, the cause of one in five deaths among men and one in six deaths among women in 2017 [10, 11]. COPD, on the other hand, was ranked as fifth among the top 10 causes of death in Finland in 2017 [11]*.*

The challenges related to using register data for determining prevalences are somewhat different for IHD and COPD. Regarding IHD, the Finnish special health care registers cover persons with IHD fairly well as most cases have specialist outpatient visits or hospital visits in long-term follow-up. Furthermore, a special reimbursement right of medicines exists for IHD. For persons without this right, the IHD diagnosis cannot be reliably deduced on the basis of medicines used by them as many medicines such as antihypertensive and cholesterol lowering medicines are used for many other purposes as well. Nitrates are, in contrast, almost exclusively used for IHD. However, nitrates are now mainly used among patients with severe conditions and multimorbidities. Those patients are very likely to have the reimbursement right for IHD.

For COPD, many medicines are currently used for both COPD and asthma, yet previously long-acting muscarinic receptor antagonists (LAMAs) were used mainly for COPD. On the other hand, mainly severe COPD patients or patients treated primarily for other diseases but who may have milder COPD as a secondary diagnosis are treated in hospitals in Finland.

This study is a part of a research project ‘Projections of the burden of disease and disability in Finland’ [12]. Two diseases, namely IHD and COPD were selected to represent diseases, which both have a considerable public health importance but different kind of issues related to availability and coverage of health data. Our aim was to estimate the burden of IHD and COPD in Finland in 2012, and to examine, how much the YLD and thereby the DALYs are affected by the use of different register data sources or population-based HES data.

## Methods

### Data sources

Abundant data sources were utilized. Several national Finnish administrative register data sources were used in YLL and YLD calculations, whereas self-reported data from a population-based HES were used to complement register data sources in YLD calculations.

### Health examination survey data

The sample used in this study comes from the National FINRISK Study, which has been described in detail earlier [13]. Shortly, the National FINRISK Study is series of cross-sectional population-based risk factor monitoring surveys, which were carried out at 5-year intervals between 1972 and 2012. In the present study, data from the FINRISK 2012 survey were used. Data were completed by comprehensive register information linked to the survey, which made it possible to compare information received from different data sources.

In 2012, a stratified random sample of 10,000 persons aged 25–74 years covering five regions in Finland was drawn from the population register. The five regions represent different geographical areas in Finland, but do not cover the whole Finnish population. The invitees received a self-administered questionnaire together with an invitation to a health examination visit. In this study, all those participants who filled in and returned the self-administered questionnaire, either by mail or during the health examination visit, were included (3041 males and 3383 females with respective participation rates 61 and 68%) (Table 1).

**Table 1:** Data characteristics of respondents in FINRISK 2012 survey^a^

	Males	Females
Participation, n (response rate %)	3041 (61%)	3383 (68%)
Age, mean (SD)	51.8 (14.0)	50.4 (14.1)
**Survey questionnaire responses**		
Effort angina (angina pectoris)^b^	4.6%	2.1%
Myocardial infarction ever	3.6%	0.9%
Coronary bypass surgery ever	2.2%	0.3%
Coronary angioplasty ever	3.1%	0.6%
Chronic obstructive pulmonary disease (COPD)^b^	1.6%	0.7%

*SD* Standard deviation

^a^All figures are unadjusted

^b^The disease was included in a list of diseases under a question: Have you suffered or received treatment for any of the following diseases (diagnosed by a doctor) during the past year (last 12 months)?

Self-reported information on IHD and COPD was based on responses to the survey questionnaire. The questions on IHD were: 1) Have you suffered or received treatment for angina pectoris (diagnosed by a doctor) during the past year, 2) Have you ever been diagnosed by a doctor for myocardial infarction, 3) coronary bypass surgery ever, or 4) coronary angioplasty ever. The question on COPD was 1) Have you suffered or received treatment for chronic obstructive pulmonary disease (COPD) (diagnosed by a doctor) during the past year. A positive response in any of these questions was considered as subject having IHD or COPD, respectively.

### Register data

For YLL calculations, mortality data for the whole of Finland in 2012 (January 1st to December 31st, 2012) were obtained from the StatFin database of the Statistics Finland, which is an open database [14]. For the current study, the number of deaths with IHD or COPD as the underlying cause of death among the Finnish population was retrieved separately for males and females. The mortality data are cause-specific only and do not include any estimates of ill-defined deaths that would be redistributed to IHD and COPD. The International Classification of Diseases, 10th revision (ICD-10) codes I20–I25 were used for IHD and codes J41–J44 for COPD. These are the same ICD-10 codes for IHD and COPD that were applied in the GBD 2017 study [1, 2]. The mortality data were available in 5-year age categories. Because the survey data were limited to age group 25–74 years, data on IHD and COPD mortality for the same age groups were used in the calculations of YLL. However, mortality data for all age groups, including those not analyzed by us, are presented for informational purposes in Additional file 1.

**Fig. 1:**
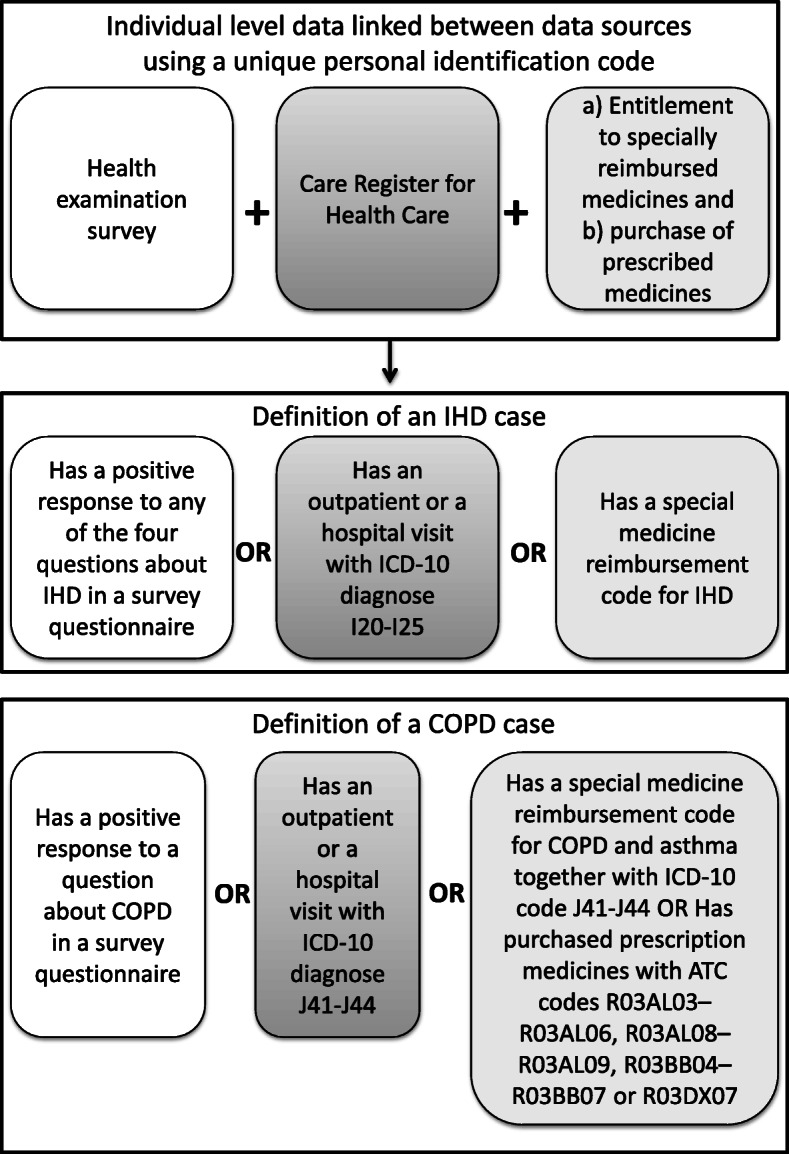
Data sources for YLD. YLD, years lived with disability; IHD, ischemic heart disease; ICD, International Classification of Diseases; COPD, chronic obstructive pulmonary disease; ATC, Anatomical Therapeutic Chemical

For YLD calculations, data from several registers were linked with the FINRISK survey data using the unique personal identification code (PIC) of the residents in Finland. All register records that preceded the date of each participant’s survey participation were retrieved. All persons with a record attesting that the person had been diagnosed with either IHD or COPD were classified as prevalent disease cases irrespective of the severity level of the disease. The data sources are illustrated in Fig. 1.

The two register sources used for estimating the YLD of IHD were 1) the Care Register for Health Care (a continuation of the Hospital Discharge Registry) including hospital inpatient episodes and specialist outpatient visits, and 2) entitlement to specially reimbursed medicines from the Registers of the Social Insurance Institution of Finland. For COPD, additionally information on purchases of prescribed medicines by the Anatomical Therapeutic Chemical (ATC) Classification System code from the Registers of the Social Insurance Institution of Finland was used.

The Care Register for Health Care covers the health care records of inpatients episodes in all hospitals since 1969, inpatient episodes in municipal health centers since 1994 and specialist outpatient visits since 1998 in Finland. The Care Register for Health Care includes one or several diagnosis codes associated with each episode up to a maximum of six diagnosis codes. All persons who had any register record with ICD-10 code related to IHD or COPD were classified as having the condition. The same ICD-10 codes were applied as in the case of mortality data (I20–I25 for IHD and J41–J44 for COPD).

Information on the entitlement to specially reimbursed medicines from the Registers of the Social Insurance Institution of Finland is available since 1964. For IHD, there is a separate medicine reimbursement code. Persons with entitlement to specially reimbursed medicines on the basis of having IHD could be identified according to IHD’s reimbursement code. The criterion for IHD reimbursement status is that a patient has clinically approved chronic angina pectoris responding to medication, a patient has had a myocardial infarction or severe constriction of coronary arteries which has been confirmed in angiography or a patient has had invasive treatments such as bypass surgery or angioplasty. In the current study, a person was classified as having IHD if the entitlement had started before the date of the participant’s survey visit. In the case of COPD, the Finnish medical reimbursement system covers medicines for severe or very severe COPD with a criterion of forced expiratory volume per 1 sec (FEV1) below 40% after bronchodilation or FEV1 below 50% and either one exacerbation treated in hospital or at least two per oral treatments with corticosteroid prescribed for COPD exacerbation, corresponding closest to the international Global Initiative for Chronic Obstructive Lung Disease (GOLD) stages 3 and 4 [15]. In practice, using register data on the entitlement to specially reimbursed medicines to identify COPD cases is complicated by the coding system, as COPD shares a common medicine reimbursement code with asthma. The ICD code for the disease for which the reimbursement was entitled has been gradually added to the register in connection to the reimbursement code with moderate coverage since 2003. Thereby, persons with these two records in the Registers of the Social Insurance Institution of Finland, the common reimbursement code for asthma and COPD and an ICD code record indicating that the reimbursement was due to COPD could be classified as having COPD. Again, the same ICD-10 codes were applied for COPD as for other registers (J41–J44), but as explained above, the entitlement was possible only in the case of severe COPD.

Information on purchase of prescribed medicines by ATC code from the Registers of the Social Insurance Institution of Finland was derived to complement register data sources for COPD and is reported combined with data on the entitlement to specially reimbursed medicines. The data on medicine purchases are available since 1995, and this register includes all medicines prescribed by a physician and purchased by the patient. However, most pulmonary medicines are not uniquely used for COPD and treatment guidelines for COPD have changed during last 20 years, and therefore, the medicine purchase data was not an optimal data source. In this study, the selected ATC codes included medicines which were mainly used for COPD before 2012 and available in Finland*.* The ATC codes included were R03AL03–R03AL06 (combined inhaled long-acting B2-sympatomimetics and muscarinic receptor antagonists medication), R03AL08–R03AL09 (combined inhaled long-acting B2-sympatomimetics, long acting LAMA and steroid medication), R03BB04–R03BB07 (long-acting inhaled LAMA medications) and R03DX07 (rofrumilast).

### YLL, YLD and DALY calculations

DALYs for IHD and COPD were calculated without age-weighting or time discounting and YLD were estimated using prevalence-based approach in concordance with GBD 2016 analyses [1]. All calculations were performed separately for men and women. Since the survey data covered participants aged 25–74 years, all analyses were limited to this age group.

### Years of life lost due to premature mortality (YLL)

The YLL were calculated by multiplying the number of deaths in a given age group with the standard expected years of life lost (SEYLL) from the WHO Standard Life Table for Years of Life Lost [1]. We used the SEYLL for the midpoint of each 5-year age group in the YLL calculations. As an example, for the age group 25–29 years the SEYLL of 27-year-olds (65.09 years) were used. The YLL in all included age groups (25–74 years) were then summed up separately for males and females. In addition to the absolute numbers of YLL, the YLL per 1000 population were calculated using the 25–74-year-old population in Finland in the end of year 2012 (1,707,636 males and 1,714,309 females).

### Years lived with disability (YLD)

YLD were calculated using the FINRISK 2012 survey data (described above) [13]. The prevalences were calculated separately for the included data sources (two or three national administrative registers and self-reported data from the FINRISK survey) and weighted using survey weights to represent the 25–74-year-old population in the study areas in 2012 and to adjust for the survey non-participation. The weighted prevalences were then directly adjusted to the whole Finnish population aged 25–74 in 2012 using population statistics from the Statistics of Finland, which resulted in the estimated numbers of males and females with the conditions in Finland [16]. The numbers of persons were then multiplied with the weighted disability weights (see below) to calculate the absolute number of YLD in Finland in 2012. The YLD per 1000 population were also calculated.

### DALY calculation

For the final DALY figures, the YLL and YLD were summed up. In addition to the absolute numbers of DALYs, the DALYs per 1000 population were calculated.

### Disability weights

We used disability weights from the WHO GBD 2017 study [1, 2]. As there are disability weights for different severity levels of IHD and COPD, we calculated weighted disability weights for the two conditions based on published severity distributions [3]. We weighted the four disability weights for the four severity levels (asymptomatic, mild, moderate and severe) with the estimated proportion of persons with the condition in question belonging to that severity level group. We did not stratify the conditions into sub-groups with and without heart failure. The resulting weighted disability weights were 0.073 for IHD and 0.127 for COPD.

## Results

### Ischemic heart disease

The results for YLL, YLD and DALYs for IHD are presented per 1000 population in Table 2. The results for corresponding absolute figures and according to a more thorough classification by register sources as well as including different combinations of data sources are presented in Additional file 2.

**Table 2:** Prevalences and YLL, YLD and DALYs for IHD in Finland per 1000 population using administrative health register and self-reported survey data from the FINRISK 2012 survey

	The Care Register for Health Care^a^	Entitlement to specially reimbursed medicines^b^	All registers combined	Self-reported data (HES)	All data sources combined
**Males**					
YLL per 1000 = 37.5					
Prevalence (%)	4.8	3.6	4.9	6.2	7.3
YLD per 1000	3.5	2.7	3.6	4.5	5.3
DALYs per 1000	**40.9**	**40.1**	**41.0**	**42.0**	**42.8**
**Females**					
YLL per 1000 = 9.1					
Prevalence (%)	2.0	1.4	2.2	2.4	3.4
YLD per 1000	1.4	1.0	1.6	1.8	2.5
DALYs per 1000	**10.6**	**10.1**	**10.7**	**10.9**	**11.6**

*YLL* Years of life lost, *YLD* Years lived with disability, *DALYs* Disability-adjusted life years, *IHD* Ischemic heart disease, *HES* Health examination survey

^a^The Care Register for Health Care includes hospital inpatient episodes since 1969 and specialist outpatient visits since 1998

^b^From the Registers of the Social Insurance Institution of Finland

The YLL for IHD were 37.5 for males and 9.1 for females per 1000 population among 25–74-year-old people in Finland in 2012 (Table 2).

The HES data resulted in higher IHD prevalence than all included registers together (6.2% vs. 4.9 and 2.4% vs. 2.2% for males and females, respectively), and combining register data and HES data elevated the prevalence even further (to 7.3% for males and 3.4% for females) (Table 2). Thereby, the YLD varied markedly depending on which data sources were used to estimate the prevalence. All data sources combined, the YLD per 1000 population were 5.3 for males and 2.5 for females.

**Fig. 2:**
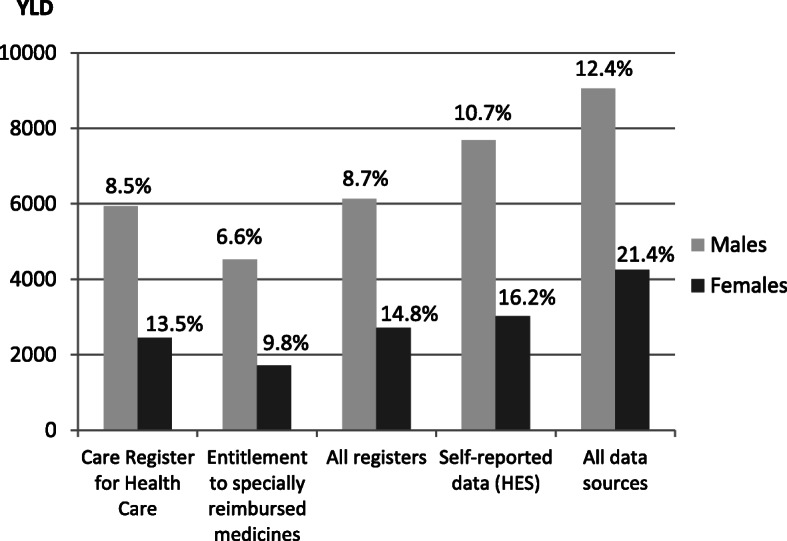
Total YLD for IHD in Finland in 2012 and the proportion of YLD of DALYs^1^. YLD, years lived with disability; IHD, ischemic heart disease; DALYs, disability-adjusted life years; HES, health examination survey. ^1^The percentages of YLD of DALYs are presented above each bar

The YLD constituted a minor part of total DALYs compared to YLL. However, the contribution of YLD to total DALYs varied considerably depending on which data sources were used to calculate YLD (6.6–12.4% and 9.8–21.4% among males and females, respectively) (Table 2, Fig. 2).

Using data from all three registers and from HES resulted in estimated 42.8 DALYs per 1000 population and 11.6 DALYs per 1000 population due to IHD among males and females, respectively (Table 2). The DALYs increased by about four and 10 % for males and females, respectively, when data from all sources instead of data from the Care Register for Health Care only were used. The DALYs for IHD were about four-fold among males compared to females.

The absolute number of DALYs due to IHD was 73,017 for males and 19,916 for females (Additional file 2).

### Chronic obstructive pulmonary disease

The results for YLL, YLD and DALYs for COPD are presented per 1000 population in Table 3. The results for corresponding absolute figures and according to a more thorough classification by register sources as well as including different combinations of data sources are presented in Additional file 3.

**Table 3:** Prevalences and YLL, YLD and DALYs for COPD in Finland per 1000 population using administrative health register and self-reported survey data from the FINRISK 2012 survey

	The Care Register for Health Care^a^	Registers of the Social Insurance Institution^b^	All registers combined	Self-reported data (HES)	All data sources combined
**Males**					
YLL per 1000 = 4.7					
Prevalence (%)	0.8	0.6	1.1	1.2	1.6
YLD per 1000	1.0	0.8	1.3	1.5	2.0
DALYs per 1000	**5.7**	**5.5**	**6.0**	**6.2**	**6.7**
**Females**					
YLL per 1000 = 2.0					
Prevalence (%)	0.5	0.7	0.9	0.8	1.3
YLD per 1000	0.6	0.9	1.1	1.0	1.6
DALYs per 1000	**2.6**	**2.9**	**3.1**	**3.0**	**3.6**

*YLL* Years of life lost, *YLD* Years lived with disability, *DALYs* Disability-adjusted life years, *COPD* Chronic obstructive pulmonary disease, *HES* Health examination survey

^a^The Care Register for Health Care includes hospital inpatient episodes since 1969 and specialist outpatient visits since 1998

^b^Data on the 1) entitlement to specially reimbursed medicines and 2) purchase of prescribed COPD medicines from the Registers of the Social Insurance Institution of Finland combined

The YLL for COPD were 4.7 for males and 2.0 for females per 1000 population among 25–74-year-old people in Finland in 2012 (Table 3).

The different register sources resulted in substantial differences in the estimated COPD prevalence. HES data resulted in quite similar prevalence estimates as all included registers together (1.2% vs. 1.1 and 0.8% vs. 0.9% for males and females, respectively), but combining both register data and HES data elevated the prevalence estimates considerably (to 1.6% for males and to 1.3% for females) (Table 3). The YLD per 1000 population based on all data sources combined were 2.0 for males and 1.6 for females.

**Fig. 3:**
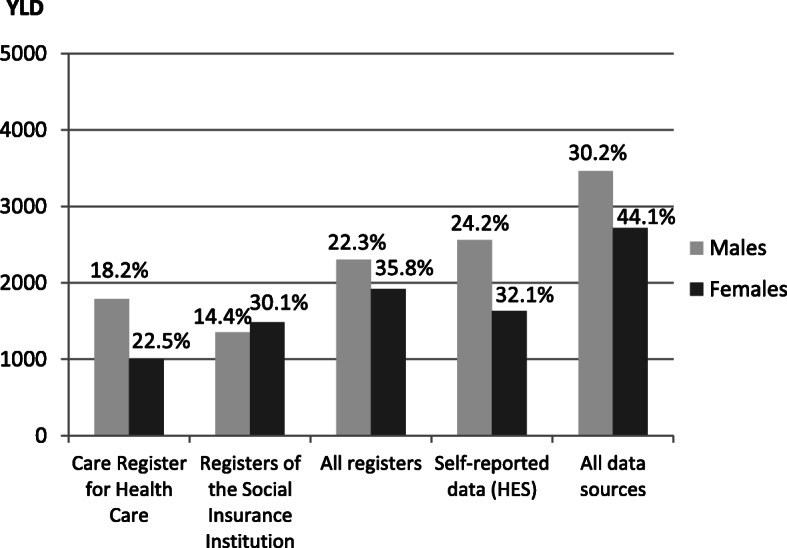
Total YLD for COPD in Finland in 2012 and the proportion of YLD of DALYs^1^. YLD, years lived with disability; COPD, chronic obstructive pulmonary disease; DALYs, disability-adjusted life years; HES, health examination survey. ^1^The percentages of YLD of DALYs are presented above each bar

The proportion of YLD of total DALYs varied considerably depending on data sources selected to calculate YLD (14.4–30.2% and 22.5–44.1% among males and females, respectively) in accordance with the variation of the observed prevalences by data source (Table 3, Fig. 3).

Combining data from all sources resulted in estimated 6.7 and 3.6 DALYs per 1000 population due to COPD in Finland in 2012 among men and women, respectively (Table 3). The DALYs increased by about 17 and 38% for males and females, respectively, when data from all sources instead of data from the Care Register for Health Care only were used. The DALYs for COPD were almost two-fold among males compared to females.

The absolute number of DALYs due to COPD was 11,472 for males and 6166 for females (Additional file 3).

## Discussion

Using several instead of single register sources resulted in higher prevalence estimates for IHD and even more so for COPD. Moreover, using HES data in addition to register data increased the prevalence rates and thereby the YLD and DALY estimates, which we believed to improve the accuracy of the final figures. More than 90,000 DALYs were caused by IHD and almost 18,000 by COPD in Finland in 2012. Per 1000 population, these figures correspond to 43 DALYs for males and 12 DALYs for females for IHD and 7 DALYs for males and 4 DALYs for females for COPD. Premature mortality (i.e. YLL) accounted for a major part of the DALYs for IHD, whereas for COPD, also morbidity (i.e. YLD) caused substantial burden.

The figures in this study are calculated based on observed data on individual level from several separate data sources, which included both register data and self-reported data from a population-based HES. Yet, our study has several limitations, especially with respect to the study material. Only the age group 25–74 years was included, as we have HES data only for this age group. However, as can be observed from the Additional file 1, the number of both IHD and COPD deaths increase after 75 years of age. Thereby, the upper age limit of 75 excludes a significant number of IHD and COPD cases. However, the SEYLL are small in the oldest age groups, and estimating the final effect of exclusion of the over 75-year-old population is not simple. In a study from the United States, the YLL, YLD and DALYs for myocardial infarction and COPD were actually lower among over 80-year-olds than in the 10-year age groups between 50 and 79 years [17]. By contrast to the exclusion of the elderly age groups, setting the lower age limit to 25 years instead of birth was likely to have a negligible effect on the results in this study. In future studies, the results of this study might be used for extrapolation to include older age groups to the DALY estimations or even same age groups as in our study but where data sources are less abundant.

We used the GBD Results Tool [18] and compared our results to those for the same age groups in Finland in 2012. Our YLD estimates were higher for IHD and lower for COPD than those used in the GBD study. We were able to combine individual level health data from different sources, whereas the Institute for Health Metrics and Evaluation (IHME) uses aggregated data and results published in scientific literature to estimate burden of disease metrics. This may have allowed us to obtain more precise estimates, since we did not have to rely on so many modelling assumptions.

Our survey sample only included survey participants, i.e. those who at least filled in and returned the survey questionnaire. However, non-participants are likely to be younger, less well educated, non-married, smokers, and have a less favourable health status and higher mortality in general [19–23]. In our study, we used survey weights to account for the effect of non-participation bias with respect to age, sex and area. However, it is possible that non-participation depended on the outcomes of interest, thus the prevalences might be underestimated.

Also the register data contains possible flaws and sources of error. Regarding the calculation of YLL, we relied on mortality data from Statistics Finland. Ill-defined deaths in mortality data might have caused inaccuracy in the number of IHD or COPD deaths and thereby led to inaccuracy in our YLL estimates. This inaccuracy is likely to be very small, as the Finnish national legislation and practices in cause of death investigation aim at a very accurate cause of death determination and the percentage of “garbage coded” deaths in Finland has been estimated to be only 5 % [24, 25].

For YLD, one of the limitations is that our data does not cover visits to primary health care units. Unfortunately, register data on primary care are available with a good coverage in Finland only since 2013 while we used register records until each person’s survey visit during January–May 2012. In future studies, data from primary health care units would, however, evidently improve the coverage of register data especially for COPD, as persons with milder or asymptomatic COPD do not necessarily visit special health care units.

By contrast, the Care Register for Health Care (a continuation of the Hospital Discharge Registry) has a long history of systematic data collection, and has been widely utilized for health monitoring and research purposes in Finland [6, 7]*.* For IHD events, the diagnoses of fatal and non-fatal IHD events in the Hospital Discharge Register and Causes of Death register were estimated to be reasonably valid already more than 15 years ago [26]. More recently, the validity of heart failure diagnoses in the Hospital Discharge Register, ie. the Care Register for Health Care, was examined, with a conclusion that for this condition, the register data were reliable [27].

Several issues complicate the use of register data in identifying COPD cases. Firstly, in case that a COPD patient meets the diagnostic criteria for asthma, the disease may in many cases have been registered as asthma for medical reimbursement. Asthma as a diagnosis is also less stigmatized for the patient. Secondly, COPD and asthma share the same code for the entitlement to specially reimbursed medicines from the Registers of the Social Insurance Institution of Finland, and it was not possible to differentiate between the two diseases in older data of this register where no ICD code was recorded in connection to the entitlement code. Thus, we could only utilize the more recent records from this register. In addition, the entitlement to specially reimbursed medicines for COPD is only possible for severe COPD. Therefore, we complemented COPD data with the register on purchases of prescribed medicines. Some subjects who may not have an entitlement to specially reimbursed medicines but use medicines for the condition may have been identified based on their medicine purchases.

Undoubtedly, our data sources do not cover all diagnosed COPD cases, let alone persons who have not been diagnosed and are unaware of their COPD status. To achieve reliable data on COPD, spirometry and bronchodilator test should be included in the survey protocol as has been done in some surveys. In Finland, the Health 2000 and Health 2011 surveys included spirometry [28, 29]. A post-bronchodilator test finding suggesting airway obstruction (forced expiratory volume per 1 sec (FEV1) / forced vital capacity (FVC) < 70%) [15] was observed in 11% of males and 6% of females in 2000 and 13 and 9% in 2011. The Health 2000 and Health 2011 surveys included participants who were at least 30 years old with no upper age limit. In both surveys, the prevalence of suggestive airway obstruction increased markedly with age, although it should be acknowledged that the cut-off (FEV1/FVC < 70%) leads to overestimates among the elderly [30]. For working age population only (30–64 years) in 2000, the prevalence was substantially lower than when the older participants were included: 6% for males and 3% for females [28].

Unfortunately, it was not possible to adequately estimate the actual severity level of IHD and COPD from the registers or HES data to apply the different disability weights for each severity level of these diseases. Therefore, we applied the severity level distributions estimated for the GBD study for IHD and COPD [3]. It should be borne in mind, however, that the severity distributions have been estimated on the basis of two studies from the United States and one from Australia, and may not ideally reflect the severity distributions in Finland [31]. In Scotland, national weighted disability weights have been calculated for 21 cancer types based on local severity distributions and compared to disability weights weighted using GBD 2016 global severity distributions [32]. The severity distributions used in GBD 2016 study differed markedly from the Scottish national severity distributions with higher disability weight estimates in a large majority of the selected cancer types. Furthermore, assuming that all asymptomatic or milder COPD cases could not be identified from our data sources, the severity distributions that were used might not reflect the severity distributions of our data. In comparison to using only register data, including self-reported data probably helped to identify less severe cases as well.

Assuming that we were not able to identify all asymptomatic or milder COPD cases, we performed a sensitivity analysis and upscaled the prevalences according to the proportions of estimated severity distributions [3]. The calculation was based on an assumption that only severe or moderate cases were identified from our data sources. These upscaled prevalence rates were about three-fold compared to those that were calculated applying the severity distributions directly to our actual data (Additional file 4), and led to considerably higher proportions of YLD of DALYs (for males 55.3% and for females 69.3% compared to 30.2 and 44.1%, respectively, including all data sources). In Scotland, it has been estimated that 31% of disease burden due to COPD in 2015 was from morbidity [33], which is more similar with our data without upscaling the prevalence rates.

For the two conditions examined in this study but even more so for IHD, YLL, which rely on mortality statistics, compose a major part of DALY. Thereby, the variation in morbidity estimates (i.e. YLD) depending on included data sources only led to moderate differences in DALYs. This can also be illustrated by quantifying how many additional persons suffering from IHD/COPD in the whole Finnish population in 2012 would have led to a 1 % increase in DALYs. This was about 10,000 and 2700 IHD cases and 900 and 490 COPD cases for men and women, respectively.

There was considerable variation in the prevalence rates and thereby the YLD estimates of IHD and COPD when the results were reported separately for each individual data source. As discussed above, all disease cases may not be identified from the registers. Self-report, on the other hand, may include overestimates, e.g. due to possible misinterpretation of symptoms, such as assuming dyspnea caused by other reasons to be heart disease related. Combining information from several data sources was, however, likely to improve the final DALY estimates. Overall, the effect of variation in YLD estimates on DALYs would probably be larger in the case of diseases, which cause more morbidity in relation to premature mortality than IHD and COPD.

## Conclusions

The different combinations of data sources markedly affected the YLD of IHD and COPD. However, as YLL constitute a major part of DALYs for these diseases, the variation in YLD did not lead to substantial variation in DALYs.

## Supplementary information

**Additional file 1.** Deaths in ischemic heart disease (IHD) (ICD-10 codes I20–I25) and COPD (chronic obstructive pulmonary disease) (ICD-10 codes J41–J44) in Finland in 2012 (January 1st to December 31st) by the underlying cause of death and the population of Finland (December 31st, 2012) and by 5-year age groups. Description of data: Deaths in ischemic heart disease and COPD for all age groups in Finland, 2012

**Additional file 2.** Prevalences and YLL, YLD and DALYs for IHD in Finland using administrative health register and self-reported survey data from the FINRISK 2012 survey for all data sources separately and for different combinations of data sources (3041 males, 3383 females). Description of data: YLL, YLD and DALYs for IHD in absolute figures and per 1000 population by data sources and including different combinations of data sources

**Additional file 3.** Prevalences and YLL, YLD and DALYs for COPD in Finland using administrative health register and self-reported survey data from the FINRISK 2012 survey for all data sources separately and for different combinations of data sources (3041 males, 3383 females). Description of data: YLL, YLD and DALYs for COPD in absolute figures and per 1000 population by data sources and including different combinations of data sources

**Additional file 4.** Sensitivity analysis with upscaled prevalences and YLL, YLD and DALYs for COPD in Finland per 1000 population using administrative health register and self-reported survey data from the FINRISK 2012 survey. Description of data: Sensitivity analysis with upscaled prevalances for COPD

## Data Availability

Mortality data are available from the StatFin database of the Statistics Finland. The database is publicly accessible on the web page of the Statistics Finland (http://pxnet2.stat.fi/PXWeb/pxweb/en/StatFin/). Also the data on the population of Finland in 2012 was derived from the StatFin database of the Statistics Finland. The survey datasets and linked register datasets analysed in the current study are not publicly available due to restrictions based in the General Data Protection Regulation (GDPR) on sensitive data such as personal health data. The access to the data for research collaboration may be requested through the Finnish Institute for Health and Welfare (THL) Biobank (https://thl.fi/en/web/thl-biobank/for-researchers).

## Abbreviations

ATC: Anatomical Therapeutic Chemical
COPD: Chronic obstructive pulmonary disease
DALY: Disability-adjusted life year
FEV1: Forced expiratory volume per 1 sec
FVC: Forced vital capacity
GBD Study: The Global Burden of Disease Study
GOLD: The Global Initiative for Chronic Obstructive Lung Disease
HES: Health examination survey
ICD: International Classification of Diseases
IHD: Ischemic heart disease
IHME: Institute for Health Metrics and Evaluation
LAMAs: Long-acting muscarinic receptor antagonists
PIC: Personal identification code
SEYLL: Standard expected years of life lost
YLD: Years lost due to disability
YLL: Years of life lost
