# Supplementary material for: Comparing data sources in estimating disability-adjusted life years (DALYs) for ischemic heart disease and chronic obstructive pulmonary disease in a cross-sectional setting in Finland
Source: Arch Public Health. 2020 Jun 18;78:58. doi: 10.1186/s13690-020-00439-6 (PMC7302348; doi:10.1186/s13690-020-00439-6)
Supplement: Supplementary file 1 — Additional file 1. Deaths in ischemic heart disease (IHD) (ICD-10 codes I20–I25) and COPD (chronic obstructive pulmonary disease) (ICD-10 codes J41–J44) in Finland in 2012 (January 1st to December 31st) by the underlying cause of death and the population of Finland (December 31st, 2012) and by 5-year age groups. Description of data: Deaths in ischemic heart disease and COPD for all age groups in Finland, 2012 [file 13690_2020_439_MOESM1_ESM.pdf]

Additional file 1. Deaths in ischemic heart disease (IHD) (ICD-10 codes I20–I25) and chronic obstructive pulmonary disease (COPD) (ICD-10 codes J41–J44) in Finland in 2012 (January 1st to December 31st) by the underlying cause of death and the population of Finland (December 31st, 2012) and by 5-year age groups

	**IHD deaths 2012**		**COPD deaths 2012**		**Population**	
Age group	Males	Females	Males	Females	Males	Females
0–24	0	0	0	0	792 752	759 111
25–29	1	0	0	0	175 226	166 069
30–34	2	1	0	0	177 454	167 305
35–39	6	2	0	0	169 502	160 340
40–44	22	6	1	0	166 649	160 821
45–49	48	8	1	2	188 707	184 591
50–54	139	16	6	3	185 863	185 308
55–59	266	37	23	14	187 478	191 717
60–64	480	97	66	17	191 222	198 366
65–69	614	169	89	46	158 967	172 561
70–74	676	258	118	48	106 568	127 231
75–79	869	466	140	59	78 139	105 940
80–84	1 103	990	192	79	53 901	91 302
85–89	993	1 451	121	50	25 782	59 277
90–94	471	1 189	47	24	7 191	24 173
95–	142	577	6	3	1 221	5 940
**Total**	**5 832**	**5 267**	**810**	**345**	**2 666 622**	**2 760 052**

IHD, ischemic heart disease; ICD, International Classification of Diseases; COPD, chronic obstructive pulmonary disease
