# Supplementary material for: Comparing data sources in estimating disability-adjusted life years (DALYs) for ischemic heart disease and chronic obstructive pulmonary disease in a cross-sectional setting in Finland
Source: Arch Public Health. 2020 Jun 18;78:58. doi: 10.1186/s13690-020-00439-6 (PMC7302348; doi:10.1186/s13690-020-00439-6)
Supplement: Supplementary file 2 — Additional file 2. Prevalences and YLL, YLD and DALYs for IHD in Finland using administrative health register and self-reported survey data from the FINRISK 2012 survey for all data sources separately and for different combinations of data sources (3041 males, 3383 females). Description of data: YLL, YLD and DALYs for IHD in absolute figures and per 1000 population by data sources and including different combinations of data sources [file 13690_2020_439_MOESM2_ESM.pdf]

Additional file 2. Prevalences and YLL, YLD and DALYs for IHD in Finland using administrative health register and self-reported survey data from the FINRISK 2012 survey for all data sources separately and for different combinations of data sources (3041 males, 3383 females)

	**Hospital inpatient episodes from the Care Register for Health Care (A)**	**Specialist outpatient visits from the Care Register for Health Care (B)**	**Entitlement to specially reimbursed medicines[1] (C)**	**A or B**	**A, B or C**	**Self-reported data (HES) (D)**	**A, B, C or D**
**Males**							
Prevalence (%)	3.8	4.1	3.6	4.8	4.9	6.2	7.3
**Total,** YLL = 63 964							
YLD	4 714	5 152	4 529	5 940	6 132	7 688	9 053
DALYs	68 678	69 116	68 493	69 904	70 096	71 652	73 017
**Per 1000 population,** YLL = 37.5							
YLD	2.8	3.0	2.7	3.5	3.6	4.5	5.3
DALYs	**40.2**	**40.5**	**40.1**	**40.9**	**41.0**	**42.0**	**42.8**
YLD of DALYs (%)	6.9	7.5	6.6	8.5	8.7	10.7	12.4
**Females**							
Prevalence (%)	1.2	1.7	1.4	2.0	2.2	2.4	3.4
**Total,** YLL = 15 662							
YLD	1 539	2 115	1 711	2 449	2 711	3 028	4 254
DALYs	17 201	17 778	17 373	18 111	18 373	18 690	19 916
**Per 1000 population,** YLL = 9.1							
YLD	0.9	1.2	1.0	1.4	1.6	1.8	2.5
DALYs	**10.0**	**10.4**	**10.1**	**10.6**	**10.7**	**10.9**	**11.6**
YLD of DALYs (%)	8.9	11.9	9.8	13.5	14.8	16.2	21.4

YLL, years of life lost; YLD, years lived with disability; DALYs, disability-adjusted life years; IHD, ischemic heart disease; HES, health examination survey

[1]From the Registers of the Social Insurance Institution of Finland
