# Supplementary material for: Comparing data sources in estimating disability-adjusted life years (DALYs) for ischemic heart disease and chronic obstructive pulmonary disease in a cross-sectional setting in Finland
Source: Arch Public Health. 2020 Jun 18;78:58. doi: 10.1186/s13690-020-00439-6 (PMC7302348; doi:10.1186/s13690-020-00439-6)
Supplement: Supplementary file 3 — Additional file 3. Prevalences and YLL, YLD and DALYs for COPD in Finland using administrative health register and self-reported survey data from the FINRISK 2012 survey for all data sources separately and for different combinations of data sources (3041 males, 3383 females). Description of data: YLL, YLD and DALYs for COPD in absolute figures and per 1000 population by data sources and including different combinations of data sources [file 13690_2020_439_MOESM3_ESM.pdf]

Additional file 3. Prevalences and YLL, YLD and DALYs for COPD in Finland using administrative health register and self-reported survey data from the FINRISK 2012 survey for all data sources separately and for different combinations of data sources (3041 males, 3383 females)

	**Hospital inpatient episodes from the Care Register for Health Care (A)**	**Specialist outpatient visits from the Care Register for Health Care (B)**	**Entitlement to specially reimbursed medicines[1] (C)**	**Purchase of prescribed COPD medicines (D)[1]**	**A or B**	**A, B or C**	**A, B, C or D**	**Self-reported data (HES) (E)**	**A, B, C, D or E**
**Males**									
Prevalence (%)	0.3	0.7	0.1	0.6	0.83	0.84	1.1	1.2	1.6
**Total,** YLL = 8 009									
YLD	564	1 508	242	1 352	1 787	1 817	2 303	2 562	3 463
DALYs	8 573	9 517	8 250	9 361	9 796	9 826	10 312	10 571	11 472
**Per 1000 population,** YLL = 4.7									
YLD	0.3	0.9	0.1	0.8	1.0	1.1	1.3	1.5	2.0
DALYs	**5.0**	**5.6**	**4.8**	**5.5**	**5.7**	**5.8**	**6.0**	**6.2**	**6.7**
YLD of DALYs (%)	6.6	15.8	2.9	14.4	18.2	18.5	22.3	24.2	30.2
**Females**									
Prevalence (%)	0.2	0.4	0	0.7	0.5	0.5	0.9	0.8	1.3
**Total,** YLL = 3 449									
YLD	399	813	0	1 487	1 003	1 003	1 920	1 634	2 717
DALYs	3 847	4 262	3 449	4 936	4 452	4 452	5 369	5 083	6 166
**Per 1000 population,** YLL = 2.0									
YLD	0.2	0.5	0	0.9	0.6	0.6	1.1	1.0	1.6
DALYs	**2.2**	**2.5**	**2.0**	**2.9**	**2.6**	**2.6**	**3.1**	**3.0**	**3.6**
YLD of DALYs (%)	10.4	19.1	0	30.1	22.5	22.5	35.8	32.1	44.1

YLL, years of life lost; YLD, years lived with disability; DALYs, disability-adjusted life years; COPD, chronic obstructive pulmonary disease; HES, health examination survey
[1]From the Registers of the Social Insurance Institution of Finland
