# Supplementary material for: Comparing data sources in estimating disability-adjusted life years (DALYs) for ischemic heart disease and chronic obstructive pulmonary disease in a cross-sectional setting in Finland
Source: Arch Public Health. 2020 Jun 18;78:58. doi: 10.1186/s13690-020-00439-6 (PMC7302348; doi:10.1186/s13690-020-00439-6)
Supplement: Supplementary file 4 — Additional file 4. Sensitivity analysis with upscaled prevalences and YLL, YLD and DALYs for COPD in Finland per 1000 population using administrative health register and self-reported survey data from the FINRISK 2012 survey. Description of data: Sensitivity analysis with upscaled prevalances for COPD [file 13690_2020_439_MOESM4_ESM.pdf]

**Additional file 4.** Sensitivity analysis with upscaled prevalences and YLL, YLD and DALYs for COPD in Finland per 1000 population using administrative health register and self-reported survey data from the FINRISK 2012 survey [1]

| | **The Care Register for Health Care[2]** | **Registers of the Social Insurance Institution[3]** | **All registers combined** | **Self-reported data (HES)** | **All data sources combined** |
|---|---|---|---|---|---|
| **Males** | | | | | |
| YLL per 1000 = 4.7 | | | | | |
| Prevalence (upscaled) (%) | 2.4 | 1.8 | 3.1 | 3.4 | 4.6 |
| YLD per 1000 | 3.0 | 2.3 | 3.9 | 4.3 | 5.8 |
| DALYs per 1000 | **7.7** | **7.0** | **8.6** | **9.0** | **10.5** |
| YLD of DALYs (%) | 39.0 | 32.6 | 45.2 | 47.8 | 55.3 |
| **Females** | | | | | |
| YLL per 1000 = 2.0 | | | | | |
| Prevalence (upscaled) (%) | 1.3 | 2.0 | 2.5 | 2.2 | 3.6 |
| YLD per 1000 | 1.7 | 2.5 | 3.2 | 2.7 | 4.5 |
| DALYs per 1000 | **3.7** | **4.5** | **5.2** | **4.7** | **6.6** |
| YLD of DALYs (%) | 45.5 | 55.3 | 61.5 | 57.6 | 69.3 |

YLL, years of life lost; YLD, years lived with disability; DALYs, disability-adjusted life years; COPD, chronic obstructive pulmonary disease; HES, health examination survey

[1]The prevalences were upscaled by assuming that our data only included moderate and severe cases and by adding the asymptomatic and mild cases according to the proportions given in the published severity distribution

[2]The Care Register for Health Care includes hospital inpatient episodes since 1969 and specialist outpatient visits since 1998

[3]Data on the 1) entitlement to specially reimbursed medicines and 2) purchase of prescribed COPD medicines from the Registers of the Social Insurance Institution of Finland combined
